# *TP53* mutations, amplification of *P63* and expression of cell cycle proteins in squamous cell carcinoma of the oesophagus from a low incidence area in Western Europe

**DOI:** 10.1054/bjoc.2001.1990

**Published:** 2001-09

**Authors:** P Tanière, G Martel-Planche, J C Saurin, C Lombard-Bohas, F Berger, J Y Scoazec, P Hainaut

**Affiliations:** Molecular Carcinogenesis, International Agency for Research on Cancer, 150 cours Albert Thomas, Lyon cedex, 69372 08, France; Service d'Anatomie Pathologique; Fédération des Spécialités Digestives, Hôpital Edouard Herriot, 1 Place d'Arsonval, Lyon cedex, 69437 03, France

**Keywords:** oesophagus, squamous cell carcinoma, low-incidence area, *TP53*, *P63*, MDM2

## Abstract

In Europe, high incidence rates of oesophageal squamous cell carcinoma (SCCE) are observed in western France (Normandy and Brittany) and in north-eastern Italy. Analysis of *TP53* mutations in tumours from these regions has shown a high prevalence of mutations at A:T basepairs that may result from DNA damage caused by specific mutagens. However, the spectrum of *TP53* mutations in regions of low incidence is unknown. We report here *TP53* mutation analysis in 33 SCCE collected in Lyon, an area of low incidence. These tumours were also examined for *MDM2* and *P63* amplification, and for expression of p16^INK4a/CDKN2a^, cyclin E, p27^Kipl^and Cox2. *TP53* mutations were detected in 36% of the cases (12/33). In contrast with regions of high incidence, the mutation spectrum did not show a high prevalence of mutations at A:T base pairs. *P63* was amplified in 5/32 cases tested (15.5%). No amplification of *MDM2* was found. Expression studies revealed frequent loss of p16^INK4a/CDKN2a^(46%) and p27^Kipl^(25%) expression, and frequent overexpression of Cyclin E (70%) and Cox2 (42%). Overall, these results indicate that in Europe, SCCE from areas of high and low incidence present a similar pattern of molecular alterations but differ by the type of *TP53* mutations. © 2001 Cancer Research Campaign http://www.bjcancer.com


British Journal of Cancer (2001) 85(5), 721–726
© 2001 Cancer Research Campaign
doi: 10.1054/ bjoc.2001.1990, available online at http://www.idealibrary.com on IDEAL

http://www.bjcancer.com

### *TP53* mutations, amplification of *P63* and expression of cell cycle proteins in squamous cell carcinoma of the oesophagus from a low incidence area in Western Europe

**P Tanière[1,2], G Martel-Planche[1], JC Saurin[3], C Lombard-Bohas[3], F Berger[2], JY Scoazec[2] and P Hainaut[1]**

[1]Molecular Carcinogenesis, International Agency for Research on Cancer, 150 cours Albert Thomas, 69372 Lyon cedex 08, France; [2]Service d'Anatomie Pathologique and [3]Fédération des Spécialités Digestives, Hôpital Edouard Herriot, 1 Place d'Arsonval, 69437, Lyon cedex 03, France

**Summary** In Europe, high incidence rates of oesophageal squamous cell carcinoma (SCCE) are observed in western France (Normandy and Brittany) and in north-eastern Italy. Analysis of *TP53* mutations in tumours from these regions has shown a high prevalence of mutations at A:T basepairs that may result from DNA damage caused by specific mutagens. However, the spectrum of *TP53* mutations in regions of low incidence is unknown. We report here *TP53* mutation analysis in 33 SCCE collected in Lyon, an area of low incidence. These tumours were also examined for *MDM2* and *P63* amplification, and for expression of $p16^{INK4a/CDKN2a}$, cyclin E, $p27^{Kipl}$ and Cox2. *TP53* mutations were detected in 36% of the cases (12/33). In contrast with regions of high incidence, the mutation spectrum did not show a high prevalence of mutations at A:T base pairs. *P63* was amplified in 5/32 cases tested (15.5%). No amplification of *MDM2* was found. Expression studies revealed frequent loss of $p16^{INK4a/CDKN2a}$ (46%) and $p27^{Kipl}$ (25%) expression, and frequent overexpression of Cyclin E (70%) and Cox2 (42%). Overall, these results indicate that in Europe, SCCE from areas of high and low incidence present a similar pattern of molecular alterations but differ by the type of *TP53* mutations. © 2001 Cancer Research Campaign http://www.bjcancer.com

**Keywords**: oesophagus; squamous cell carcinoma; low-incidence area; *TP53; P63*; MDM2

Squamous cell carcinoma of the oesophagus (SCCE) is the sixth most frequent cancer worldwide (Pisani et al, 1999). Incidence rates vary greatly between different parts of the world (for review, see Muñoz and Day, 1996). Areas of extremely high incidence are found in northern Iran (Turkoman plain) and central China (Henan province and Shanxi), where incidence rates (ASR) of over 100/100 000/year have been reported in both men and women. Other, less clearly defined high incidence areas are found in parts of South America and in south and east Africa (Muñoz and Day, 1996). In Europe and in the United States, the overall incidence does not exceed 10/100 000/year in men and 2/100 000/year in women. However, in several areas, higher incidence rates have been observed exclusively in men (up to 30/100 000/year). These areas include the north-west of France (Normandy and Brittany) and the north-east of Italy (Veneto) (Launoy et al, 1994; Parkin et al, 1997; Zambon et al, 2000). In industrialised countries, it is estimated that 90% of SCCE is attributable to tobacco and alcohol consumption, with a multiplicative effect in individuals exposed to both factors (Tuyns, 1987). In the rest of the world, a variety of cultural and dietary habits have been incriminated. These include the consumption of scalding hot beverages and a deficiency in fresh fruits and vegetables (in most areas of very high incidence), a high dietary nitrosamine content (in China and south-east Asia) and the oral consumption of opium by-products (in Northern Iran) (Muñoz and Day, 1996).

Mutation of the *TP53* gene is the most frequent genetic alteration described to date in SCCE, occurring in 35% to 70% of cancers, depending on the study and on the geographical origin of the tumours (Tanière et al, 2000b). Mutations are thought to occur at an early stage and have been observed in dysplasia, in normal mucosa adjacent to cancer lesions, and in oesophagitis in individuals from Normandy (Mandard et al, 2000). By comparing tumours from China, Thailand and western Europe, we have recently shown that the distribution and the nature of *TP53* mutations varied according to their geographic origin. This raises the possibility that the mutation pattern may provide clues on the nature of the specific mutagens involved in oesophageal carcinogenesis (Tanière et al, 2000b).

The data available on TP53 mutations in tumours from Western Europe are essentially limited to areas of high incidence of Normandy, Brittany and north-eastern Italy (Hollstein et al, 1991; Audrezet et al, 1993; Esteve et al, 1993; Li et al, 2000; Robert et al, 2000; Shirvani et al, 2000). In these areas, many mutations occur at A:T basepairs (45% of all mutations), a type of mutation which is infrequent in other cancers and in SCCE from other parts of the world. This type of mutation is consistent with DNA damage inflicted by acetaldehyde, the first metabolite of ethanol (Tudek et al, 1999). Interestingly, several studies have shown an association between a functional polymorphism in aldehyde dehydrogenase 2 (ALDH2) and the risk of SCCE, suggesting that acetaldehyde may represent an important oesophageal carcinogen (Yang et al, 1999; Aggarwal et al, 2000).

*Received 2 February 2001*
*Revised 14 May 2001*
*Accepted 15 May 2001*

*Correspondence to:* P Hainaut

BJOC 01-1990 721-726 20/8/01 3:52 pm Page 722

In the present study, we have analysed *TP53* mutations in a cohort of 33 SCCE patients from a low-incidence area of south-eastern France (Lyon, Rhône-Alpes region). In this region the reported incidence of SCCE (ASR) is 10/100 000/year in men and 1/100 000/ in women (Parkin et al, 1997). We have also analysed amplification in genes suspected to play a role in the pathogenesis of SCCE, *P63* and *MDM2*, *P63* encodes a homologue of *p53* that plays an essential role in the development of squamous epithelial. Mice lacking this gene die at birth from multiple defects due to improper skin formation (Yang et al, 1999). This gene is often amplified in primary human squamous carcinomas of the lung and the head and neck (Hibi et al, 2000; Yamaguchi et al, 2000). *MDM2* encodes a protein that binds to *p53*, inhibits its transcriptional activity and induces its degradation. Several studies have shown amplification of *MDM2* in a proportion of SCCE (14%) (Momand et al, 1998), suggesting that this phenomenon may represent a functional alternative to inactivation of *TP53* by mutation. We have also analysed by immunohistochemistry the expression of the cell cycle regulatory proteins p16$^{INK4a/CDKN2a}$, cyclin E and p27$^{Kipl}$, as well as of cyclo-oxygenase 2 (Cox2). We report that the prevalence and pattern of *TP53* mutations in this cohort differ from the ones reported in cohorts from high incidence areas of Europe. In addition, we describe for the first time that *P63* is amplified in a significant proportion of SCCE (15.3%).

### MATERIALS AND METHODS

### Patients and tumours

Tumour tissues were collected from patients recruited at Hôpital E. Herriot (Lyon, France). All patients were residents in the Lyon area. The criteria for inclusion in the study were (1) presence of a primary SCCE, (2) no primary treatment, (3) signature of an informed consent form. Tissue samples were biopsies collected during endoscopy or samples from surgical pieces. All samples were evaluated by histology. Tumour staging was performed according to the TNM classification (TNM atlas, 4th edition 1997). For biopsies, the stage of the tumour was evaluated by ultrasonography. Clinical charts were reviewed to collect information on the patient’s past medical history, tobacco and alcohol consumption, and follow-up after diagnosis and treatment. The tissue and data collection protocols were approved by local and institutional ethical committees.

### DNA extraction and *TP53* mutation detection

DNA was isolated from microdissected tissue fixed in 10% buffered formalin and embedded in paraffin. After re-hydration, areas of interest were scraped and transferred to extraction buffer (50 µl, Tris-HCl 10 mM pH 9, Proteinase K 0.1 µg ml$^{-1}$, Nonidet P40 0.1%) and incubated for 3 days at 56˚C with proteinase K. *TP53* exons 5 to 8 were analysed by temporal temperature gradient electrophoresis (TTGE) using the DCode system (BioRad, Richmond, CA) as described previously (Tanière et al, 2000a). Samples that showed additional and/or abnormal bands were re-amplified from genomic DNA and a second TTGE was performed as a confirmation. Bands corresponding to mutant alleles were cut from the second TTGE, re-amplified using the same primers and analysed by direct sequencing after asymmetric PCR as previously described (Barnas et al, 1997; Tanière et al, 2000a).

### Immunohistochemistry

Deparaffinized tissue sections were labelled using standard protocols, after antigen unmasking procedures (3 × 5 minutes in a microwave oven for slides labelled with anti-Cox2, cyclinE and p27$^{Kipl}$ antibodies, and 10 minutes in a pressure cooker for labelling with anti-mdm2). The following primary antibodies were used: CM1 (purified rabbit IgG anti-human p53, 1/500, Novocastra Laboratories Ltd, Newcastle, UK), MDM2 (Ab-1) (monoclonal antibody, clone IF2, 1/100, Calbiochem, San Diego, CA), p16$^{INK4a/CDKN2a}$ (monoclonal antibody, clone F12, 1/1600, Santa Cruz Biotechnology, Santa Cruz, CA), Cox2 (polyclonal antibody, clone C-20, 1/1000, Santacruz Biotechnology, Santa Cruz, CA), cyclin E (monoclonal antibody, clone HE-12, 1/2000, Santa Cruz Biotechnology, Santa Cruz, CA), p27$^{Kipl}$ (monoclonal antibody, clone 1B4, 1/1000, Novocastra Laboratories Ltd, Newcastle). The specificity of the antibodies was checked by Western blot using extracts of oesophageal cancer cell lines TE1, TE6 and TE11 (Barnas et al, 1997; Pluquet et al, unpublished data). Negative immunohistochemistry controls were performed by omission of the primary antibody. Fixed antibodies were detected using either anti-mouse or anti-rabbit biotinylated IgG (1/200 Vectasin Elite-ABC kit, Vector Laboratories Inc.) followed by streptavidin-peroxidase (1/50, 30 minutes at 37˚C) and diaminobenzidine-based detection (Vector Laboratories, Inc.). For Cox2 and cyclinE, tumours were considered as positive when at least 10% of tumour cells were labelled. For p27$^{Kipl}$ and p16$^{INK4a/CDKN2a}$, tumours were considered as negative when less than 10% of the tumour cells were stained.

### Amplification of *MDM2* and *P63*

Amplifications were detected by differential PCR using the dopamine D2 receptor gene (*DRD2*) as a reference (Biernat et al, 1997). Genomic DNA was amplified in 25 µl of a reaction mixture containing sense and anti-sense primers for either *MDM2* or *P63* (20 pmol) and *DRD2* (10 pmol), 200 µM of each dNTP, 1 × amplification buffer, 2.5 mM of $MgCl_2$, and 0.5 µl (2.5 units) of Taq Platinum DNA polymerase (Life Technologies). PCR conditions were: 2 minutes at 94˚C, followed by 27 cycles at 95˚C for 45 seconds, 55˚C (*MDM2*) or 53˚C (*P63*) for 45 seconds and 72˚C for 1 minute with a final extension at 72˚C for 5 minutes. The primers used in *MDM2* differential PCR were those described in Biernat et al (1997). For *P63* differential PCR, we defined the following primers in P63 exon 7 (Genbank access number AF116762): 5′-CCT ATT TGA ATT ACA TGA TGT GGA T-3′ (sense) and 5′-CAA ACT CTG AAC CCT GTT GTA GA-3′ (anti-sense). A fragment of *DRD2* with matched amplification conditions was defined with the following primers: 5′-GTT TGC TCA ATT TGT CCT ACC AG-3′ (sense) and 5′-GGG ATT TTA AGG TTT ACG GCT AA-3′ (anti-sense). For both differential PCR assays, products were electrophoresed on 3% agarose gels stained with ethidium bromide and analysed by scanning densitometry (BioRad GS-670, Hercules, CA). Each analysis was repeated at least 3 times in independent PCR experiments. A ratio of 2.5 (average of at least three measurements) or above between the specific *MDM2* or *P63* bands and the *DRD2* reference band was regarded as indicative of gene amplification.

 *© 2001 Cancer Research Campaign*

BJOC 01-1990 721-726 20/8/01 3:52 pm Page 723

### RESULTS

### Clinical and individual characteristics of the patients

The mean age of the 33 patients included in the study was 61 years (range 45–80 years). 29 were men and 4 were women. 5 patients had developed another tumour several years before the occurrence of SCCE: patient 2 (adenocarcinoma of the colon), patient 4 (malignant T-cell lymphoma), patient 19 (squamous cell carcinoma of the lung) and patients 14 and 23 (squamous cell carcinoma of the head and neck). 2 patients received a liver (patient 1) or renal (patient 9) transplant several years before SCCE. Patient 25 was surgically treated during childhood for congenital mega-oesophagus.

Reliable information on tobacco consumption was available for 23 patients. 22 (96%) were regular smokers (more than 5 pack-years). Information on alcohol intake was available for 21 patients. 17 of them were considered as heavy drinkers (daily alcohol intake over 80 g). All of these 17 patients were heavy smokers.

The pathological staging was available for all resected tumours. For patients for whom only a biopsy was available, the staging was performed by ultrasonoendoscopy. According to the TNM classification, 2 tumours were stage T1, 4 were stage T2, 21 were stage T3 and 3 were stage T4. 19 of the T3 and all T4 tumours showed lymph node involvement (N1). 2 patients (14 and 26) presented 2 distinct infiltrative lesions, and 3 patients (15 and 24) showed multiple dysplastic areas distant from the tumour.

### *TP53* mutations

A total of 13 *TP53* mutations were detected in 12/33 (36%) of the patients. These mutations are listed in Table 1. They are distributed over the exons of the DNA-binding domain (3 in exon 5, 3 in exon 6, 3 in exon 7 and 4 in exon 8). One tumour (12) contained 2 mutations. Of the 11 missense mutations, 3 were transversions and 8 were transitions, 4 of which were C to T occurring within dipyrimidine repeats.

3 mutations gave a null-*p53* phenotype (patient 1: nonsense mutation at codon 132; patients 2 and 3: 2-base pair deletion and frameshift at codons 186 and 209, respectively). These 3 tumours were negative for *p53* immunostaining. Among tumours with missense mutations, 7/9 were positive for *p53* immunostaining. Interestingly, tumours 10 and 11 showed opposite staining patterns, although they harboured the same mutation. It is also important to note that 3 tumours without mutations in exons 5–8 were found positive for *p53* immunostaining (patients 28, 29 and 31). These tumours may contain a mutation outside the regions analysed.

**Table 1** *TP53* mutations in SCCE from a low incidence area of France (Lyon)[(a)]

| Case | Codon | Base change | Amino-acid change |
|---|---|---|---|
| 1 | 132 | AAG→TAG | Tyr-Stop |
| 2 | 186 | 2 bp deletion | |
| 3 | 209 | 2 bp deletion | |
| 4 | 220 | TAT→TGT | Tyr-Cys |
| 5 | 245 | GGC→AGC | Gly-Ser |
| 6 | 245 | GGC→TGC | Gly-Cys |
| 7 | 248 | CGG→CAG | Arg-Gln |
| 8 | 272 | GTG→ATG | Val-Met |
| 9 | 272 | GTG→ATG | Val-Met |
| 10 | 273 | CGT→TGT | Arg-Cys |
| 11 | 273 | CGT→TGT | Arg-Cys |
| 12 | 135 | TGC→TGT | Cys-Phe |
| 13 | 218 | GTG→GAG | Val-Glu |

[a]Further information on sex, age and past medical history are available on: http://www.iarc.fr/p53

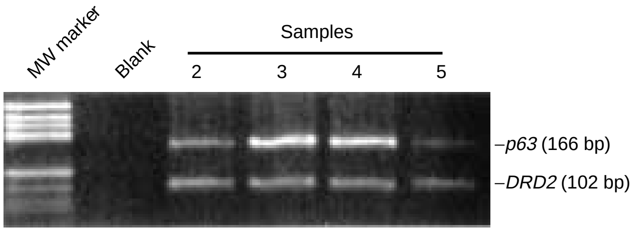

**Figure 1** Amplification of *p63* as detected by differential PCR. Tumour samples were analysed by differential PCR for *P63* gene amplification. A fragment of the *DRD2* gene was used as an internal standard. PCR products were analysed on a 3% agarose gel, stained with ethidium bromide and photographed; films were analysed by densitometry (see Materials and methods). A threshold of 2.5 was considered indicative of *P63* amplification. The intensity of the 2 bands was similar in DNA samples from normal tissue (lane 2). In tumours (lanes 3–5), the intensity of *P63* band was at least 2.5 stronger than the DRD2 band in samples 3 and 4, indicative of gene amplification. In contrast, sample 5 did not show amplification

### Amplification of *MDM2* and of *P63*

Amplification of P63 was observed in 5 of the 32 cases tested (15.5%), as detected by *P63/DRD2* gene ratios of 2.5 or above (Figure 1). 2 of these tumours also harboured a *TP53* mutation.

None of the 27 tumours tested showed *MDM2* gene amplification (Table 2). However, many tumours showed mdm2 protein expression detected by immunohistochemistry in a variable proportion of tumour cells. It is important to note that mdm2 is constitutively expressed in cells of the parabasal layers of normal oesophageal epithelium.

### Immunohistochemical detection of p16$^{INK4a/CDKN2a}$, Cox2, cyclin E and p27$^{Kip1}$

Figure 2 shows typical expression patterns for p16$^{INK4a/CDKN2a}$, Cox2, cyclin E and p27$^{Kip1}$. P16$^{INK4a/CDKN2a}$ is constitutively expressed in the basal cell layer of normal oesophageal epithelium. P16$^{INK4a/CDKN2a}$ was detectable in tumour cells in 15/28 (54%) cases tested, but lost in the remaining 13 cases (46%). 5 of these p16$^{INK4a/CDKN2a}$ negative cases also contained a *TP53* mutation. Cox2 expression was not detectable in normal oesophageal epithelium. 10/24 (42%) tumours tested were found positive for Cox2 expression (10–80% of cells stained). There was a trend of an association between positivity for Cox2 and advanced tumour stage, since only 1/7 T1, T2 and T3N0 tumours showed Cox2 expression (in 10–20% of the cells), compared with 9/17 T3N1 and T4 tumours (in at least 20% of the cells) ($P = 0.069$) (based on the 24 samples for which information on stage and Cox2 immunostaining were available, see Table 2). Cyclin E was inconstantly detectable in normal oesophageal epithelium, but was found in at least 10% of tumour cells in 16/23 (70%) cases tested, with no association with the tumour stage. Expression of p27$^{Kip1}$ was detectable in the parabasal layers of the non-involved epithelium adjacent to the tumour in all the cases. The protein was also

BJOC 01-1990 721-726 20/8/01 3:52 pm Page 724

**Table 2** Molecular alterations in SCCE patients from a low incidence area of France (Lyon)

| Case | TNM | Grade | *p53* IHC | *TP53* Mutation | *Mdm2* IHC | *MDM2* amplification | *P63* amplification |
|---|---|---|---|---|---|---|---|
| 1 | T2N0 | WD | 0 | + | > 50 | – | – |
| 2 | T3N1 | MD | 0 | + | > 50 | – | – |
| 3* | NA | PD | 0 | + | ND | ND | – |
| 4 | T1N0 | WD | > 50 | + | > 50 | ND | – |
| 5 | T2N0 | MD | 0 | + | 10–20 | – | – |
| 6* | T4N1 | WD | > 50 | + | ND | – | – |
| 7 | T3N1 | PD | 10–20 | + | 10–20 | – | – |
| 8* | T3N1 | WD | 10–20 | + | ND | – | + |
| 9* | T3N1 | WD | > 50 | + | 10–20 | – | – |
| 10 | T3N1 | WD | 0 | + | 10–20 | – | + |
| 11 | T3N1 | WD | > 50 | + | > 50 | – | – |
| 12* | T3N1 | MD | 10–20 | + | 10–20 | – | – |
| 13 | T3N1 | WD | 0 | – | 10–20 | – | – |
| 14 | T3N1 | MD | 0 | – | 20–50 | – | – |
| 15* | T4N1 | PD | 0 | – | ND | + | – |
| 16* | T3N1 | MD | 0 | – | ND | ND | – |
| 17 | T3N1 | PD | 0 | – | 0 | – | + |
| 18* | T3N1 | WD | 0 | – | > 50 | – | + |
| 19 | T2N1 | WD | 0 | – | 0 | – | + |
| 20* | T3N1 | MD | 0 | – | > 50 | – | ND |
| 21 | T4N1 | MD | 0 | – | 10–20 | – | – |
| 22* | NA | MD | 0 | – | 0 | – | – |
| 23 | NA | MD | 0 | – | > 50 | ND | – |
| 24 | T3N0 | WD | 0 | – | > 50 | – | – |
| 25 | T3N0 | WD | 0 | – | 10–20 | – | – |
| 26 | T3N1 | WD | 0 | – | 10–20 | – | – |
| 27 | T2N0 | WD | 0 | – | 20–50 | – | – |
| 28* | T3N1 | WD | > 50 | – | 20–50 | – | – |
| 29* | T3N1 | MD | > 50 | – | 10–20 | – | – |
| 30* | NA | WD | | – | ND | – | – |
| 31* | T3N1 | MD | 10–20 | – | 0 | – | – |
| 32* | T3N1 | PD | 0 | – | 0 | – | – |
| 33 | T3N1 | WD | 0 | – | ND | – | – |

*:biopsy; WD, MD, PD: well-, moderately-, poorly-differentiated; NA: not available; IHC: immunohistochemistry (percentage of stained cells in the tumour tissue); ND: not done (no more material available).

expressed in 18/24 (75%) tumours tested, with a nuclear localisation, but lost in the remaining cases (25%).

### DISCUSSION

Our results showed that *TP53* mutations in the low-incidence area of Lyon were relatively less frequent (36%) than in high-incidence areas of Europe, where mutation prevalence ranges from 56% (in northern Italy (Esteve et al, 1993) ) to 80% (in Normandy/Brittany (Audrezet et al, 1993; Robert et al, 2000) ). This rather low prevalence of mutations was not compensated by a high prevalence of *MDM2* amplification.

Despite its limited size, the present case series also showed a number of other interesting molecular characteristics. In particular, the *TP53*-related gene *P63* was amplified in 5/32 cases (15.5%). This gene has been found to be frequently amplified in squamous cell carcinomas of the lung and of head and neck and is also known as AIS (Amplified In Squamous Carcinomas) (Hibi et al, 2000). The *P63* gene encodes a transcription factor that regulates genes that overlap with those controlled by *TP53*. In contrast with *TP53*, *P63* shows a specific developmental pattern of expression and appears to be required for the normal differentiation of squamous epithelia (Yang et al, 1999). It is interesting to note that *P63* can be expressed in several isoforms (splicing variants), some of them lacking the N-terminal domain, transactivation domain. These N-terminal variants may thus behave as competitors for the full-length, active form (Yang et al, 1998). In keratinocytes, expression of such forms of *P63* is restricted to cells with high proliferative potential (Parsa et al, 1999). Such an inhibition of the antiproliferative effects of full-length *P63* may account for the oncogenic role of amplified P63/AIS. However, our data do not support the hypothesis that *P63* amplification is an alternative pathway for inactivation of *TP53*, as amplification was equally detected in tumours with wild-type (3/5) or mutant (2/5) *TP53*.

Another interesting characteristic of the series of SCCE analysed here is the association of Cox2 overexpression with advanced tumour stage. Such a positive association has been reported for many epithelial tumours, including head and neck cancers, colon cancers and both squamous cell and adenocarcinomas of the oesophagus (Ratnasinghe et al, 1999; Zimmermann et al, 1999). It is important to note that, in this study, we have considered staining in 10% of tumour cells as a threshold for positivity, a criterion more stringent than in several recent studies. Cox2 is known to enhance the synthesis of prostaglandin E2, to increase cell proliferation, angiogenesis and immune suppression and to facilitate inhibition of apoptosis. It is not known whether overexpression of Cox2 contributes to carcinogenesis or rather accompanies tumour progression as a marker of cellular stress.

Our results on $p16^{INK4a/CDKN2a}$ and $p27^{Kip1}$ expression confirm recent data showing that these 2 negative regulators of cell cycle are down-regulated in a variable proportion of SCCE (20–40%) (Itami et al, 1999; Ohashi et al, 1999; Shamma et al, 2000). In

 *© 2001 Cancer Research Campaign*

BJOC 01-1990 721-726 20/8/01 3:52 pm Page 725

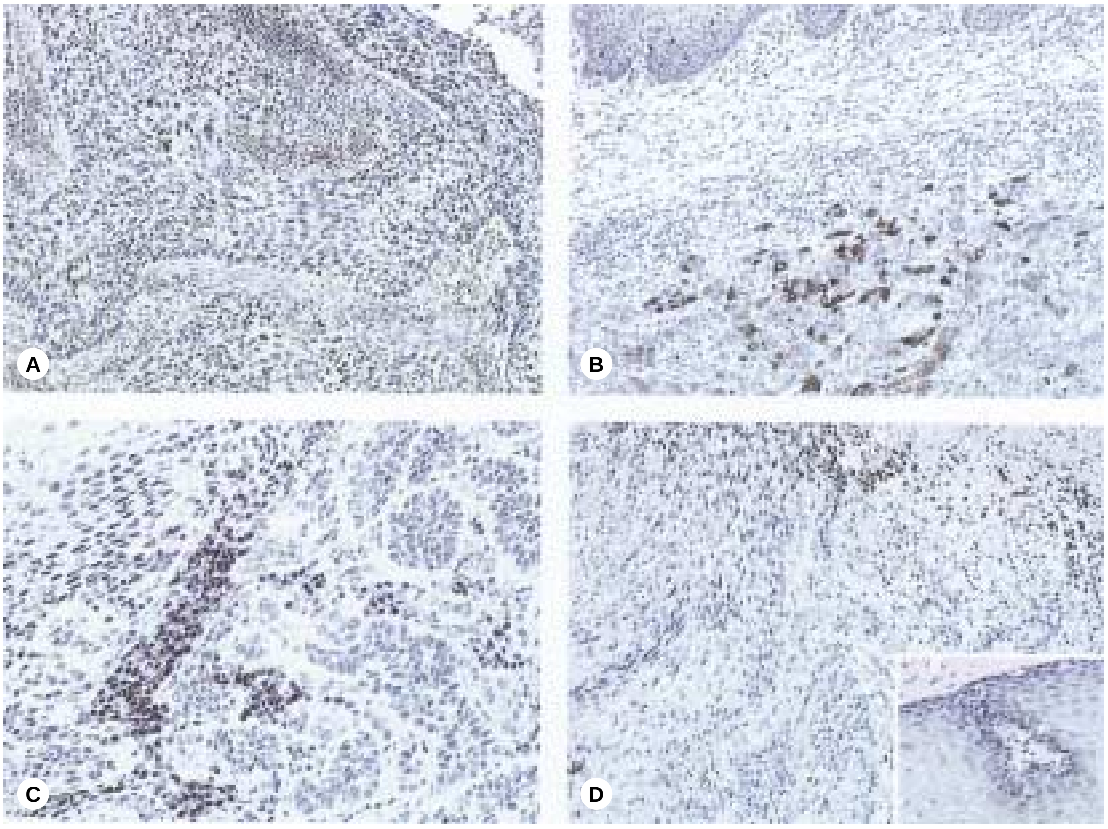

**Figure 2** Immunohistochemical detection of cyclin E (**A**), Cox2 (**B**), $p27^{Kipl}$ (**C**) and $p16^{INK4a/CDKN2a}$ (**D**) in SCCE. (**A**) More than 50% of the tumour cells were stained in the nucleus with anti-Cyclin E antibody. (**B**) 20–50% of the tumour cells expressed Cox2 in the cytoplasm. (**C**) Only 10–20% of the tumour cells expressed the $p27^{Kip1}$ protein. Lymphocytes and plasma cells were strongly stained and used as a positive control. (**D**) $p16^{INK4a/CDKN2a}$ expression was lost in tumour cells. In contrast, the non-dysplastic squamous epithelium of the oesophagus showed a focal $p16^{INK4a/CDKN2a}$ expression in the basal cell layers (insert). Scale: **A**, **B** and **D** (inset): 1 cm = 100 μM; **C** and **D**: 1 cm = 500 μM

addition, we found that most tumours (70%) expressed elevated levels of Cyclin E. High expression of Cyclin E has been reported in the majority of oesophageal cancer cell lines (Fujii et al, 1998) and in about 30% of primary SCCE (Anayama et al, 1998). Overexpression of Cyclin E has also been observed in preneoplastic lesions and in papillomas in nitrosomethylbenzylamine-induced oesophageal tumorigenesis in rats (Wang et al, 1996). Overall, these data support the observation that molecules involved in the control of cell cycle progression from G1 to S phase are often altered in SCCE. In gastric cancers, it has been shown that high levels of Cyclin E expression together with low levels of p21 and $p27^{Kip1}$, correlate with deep invasion. Such a correlation is not seen in our series of cases, as elevated Cyclin E expression was detected in 5/6 T1, T2 and T3N0 tumours, compared to 10/15 T3N1 and T4 tumours. The data available to date do not allow us to evaluate whether there are significant variations in the prevalence of these alterations between different geographic areas.

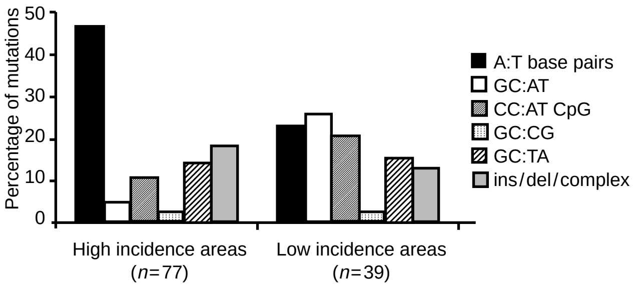

**Figure 3** Comparison between mutation patterns of primary SCCE from high- and low-incidence areas from Europe. Data reported in this article and in the published literature were used (source: IARC TP53 mutation database at http://www.iarc.fr/p53; version R5, June 2001) were used. High-incidence areas: Normandy/Brittany and northern Italy (77 mutations). Low-incidence areas: Lyon (France), Paris (France) and Lausanne (Switzerland) (39 mutations)

In many cancers, the pattern of *TP53* mutations is informative of the mutagens involved as causal agents. In Figure 3, we have grouped the data reported here with those of the IARC *TP53* mutation database. Compared with high-incidence areas, tumours from low-incidence areas show a significantly lower prevalence of transitions and transversions at A:T basepairs, a type of mutation which has been frequently detected in cancers of the oesophagus and of the head and neck, and which may result from the mutagenic action of metabolites of ethanol, such as acetaldehyde. In both high- and low-incidence areas, the main etiological agents implicated so far are the combined consumption of alcohol and tobacco (Launoy et al, 1997, 2000). However, the relative rarity of mutations at A:T basepairs in patients from low-incidence areas, most of whom were heavy drinkers, gives support to the idea that additional factors act as modifiers of the effect of alcohol. Among them, the type of oral microflora and the polymorphism of aldehyde dehydrogenase 2 (ALDH2) have been shown to be important. Indeed, high levels of acetaldehyde in the upper digestive tract are thought to derive from microbial oxidation of ethanol by the oral microflora. The composition and quantities of the oral microflora may vary from one area to another and therefore influence the actual levels of acetaldehyde that can damage the oesophageal mucosa (Muto et al, 2000b). Furthermore, it is important to note that a polymorphism in ALDH2 has been found to be associated with a higher risk of head and neck and of oesophageal cancers (Muto et al, 2000a). Whether these factors contribute to explain the variations in incidence of SCCE in Western Europe awaits further evaluation. It will also be important to evaluate whether the pattern of *TP53* mutations in other cancers related to alcohol intoxication, such as oral cancer and hepatocellular carcinomas, also vary from one geographic area to another.

### ACKNOWLEDGEMENTS

We thank JC Boulez, C Partensky and E Tissot (Hôpital Edouard Herriot) for providing surgical specimens; JA Chayvialle (Hôpital Edouard Herriot) for providing biopsies; R Montesano and J Hall for critical comments and helpful suggestions, M Olivier for analysis of the *TP53* database; N Lyandrat and M Laval for technical assistance with immunostaining and M Wrisez for secretarial assistance.

### REFERENCES

Aggarwal S, Taneja N, Lin L, Orringer MB, Rehemtulla A and Beer DG (2000) Indomethacin-induced apoptosis in esophageal adenocarcinoma cells involves upregulation of Bax and translocation of mitochondrial cytochrome C independent of COX-2 expression. *Neoplasia* **2**: 346–356

Anayama T, Furihata M, Ishikawa T, Ohtsuki Y and Ogoshi S (1998) Positive correlation between p27Kip1 expression and progression of human esophageal squamous cell carcinoma. *Int J Cancer* **79**: 439–443

Audrezet MP, Robaszkiewicz M, Mercier B, Nousbaum JB, Bail JP, Hardy E, Volant A, Lozac'h P, Charles JF and Goueron H (1993) TP53 gene mutation profile in esophageal squamous cell carcinomas. *Cancer Res* **53**: 5745–5749

Barnas C, Martel-Planche G, Furukawa Y, Hollstein M, Montesano R and Hainaut P (1997) Inactivation of the p53 protein in cell lines derived from human esophageal cancers. *Int J Cancer* **71**: 79–87

Biernat W, Kleihues P, Yonekawa Y and Ohgaki H (1997) Amplification and overexpression of MDM2 in primary (de novo) glioblastomas. *J Neuropathol Exp Neurol* **56**: 180–185

Esteve A, Lehman T, Jiang W, Weinstein IB, Harris CC, Ruol A, Peracchia A, Montesano R and Hollstein M (1993) Correlation of p53 mutations with epidermal growth factor receptor overexpression and absence of mdm2 amplification in human esophageal carcinomas. *Mol Carcinog* **8**: 306–311

Fujii S, Tominaga O, Nagawa H, Tsuno N, Nita ME, Tsuruo T and Muto T (1998) Quantitative analysis of the cyclin expression in human esophageal cancer cell lines. *J Exp Clin Cancer Res* **17**: 491–496

Hibi K, Trink B, Patturajan M, Westra WH, Caballero OL, Hill DE, Ratovitski EA, Jen J and Sidransky D (2000) AIS is an oncogene amplified in squamous cell carcinoma. *Proc Natl Acad Sci USA* **97**: 5462–5467

Hollstein MC, Peri L, Mandard AM, Welsh JA, Montesano R, Metcalf RA, Bak M and Harris CC (1991) Genetic analysis of human esophageal tumors from two high incidence geographic areas: frequent p53 base substitutions and absence of ras mutations. *Cancer Res* **51**: 4102–4106

Itami A, Shimada Y, Watanabe G and Imamura M (1999) Prognostic value of p27(Kip1) and CyclinD1 expression in esophageal cancer. *Oncology* **57**: 311–317

Launoy G, Faivre J, Pienkowski P, Milan C, Gignoux M and Pottier D (1994) Changing pattern of oesophageal cancer incidence in France. *Int J Epidemiol* **23**: 246–251

Launoy G, Milan CH, Faivre J, Pienkowski P, Milan CI and Gignoux M (1997) Alcohol, tobacco and oesophageal cancer: effects of the duration of consumption, mean intake and current and former consumption. *Br J Cancer* **75**: 1389–1396

Launoy G, Milan C, Faivre J, Pienkowski P and Gignoux M (2000) Tobacco type and risk of squamous cell cancer of the oesophagus in males: a French multicentre case-control study. *Int J Epidemiol* **29**: 36–42

Li M, Lotan R, Levin B, Tahara E, Lippman SM and Xu XC (2000) Aspirin induction of apoptosis in esophageal cancer: a potential for chemoprevention. *Cancer Epidemiol Biomarkers Prev* **9**: 545–549

Mandard AM, Hainaut P and Hollstein M (2000) Genetic steps in the development of squamous cell carcinoma of the esophagus. *Mutat Res* **462**: 335–342

Momand J, Jung D, Wilczynski S and Niland J (1998) The MDM2 gene amplification database. *Nucleic Acids Res* **26**: 3453–3459

Muñoz N and Day NE (1996). Esophageal Cancer. In: D. Schottenfeld and JF Fraumeni (eds.), *Cancer Epidemiology and Prevention* 1996, pp 681–706, Oxford University Press, Oxford, New York

Muto M, Hitomi Y, Ohtsu A, Ebihara S, Yoshida S and Esumi H (2000a) Association of aldehyde dehydrogenase 2 gene polymorphism with multiple oesophageal dysplasia in head and neck cancer patients. *Gut* **47**: 256–261

Muto M, Hitomi Y, Ohtsu A, Shimada H, Kashiwase Y, Sasaki H, Yoshida S and Esumi H (2000b) Acetaldehyde production by non-pathogenic Neisseria in human oral microflora: implications for carcinogenesis in upper aerodigestive tract. *Int J Cancer* **88**: 342–350

Ohashi Y, Sasano H, Yamaki H, Shizawa S, Shineha R, Akaishi T, Satomi S and Nagura H (1999) Cell cycle inhibitory protein p27 in esophageal squamous cell carcinoma. *Anticancer Res* **19**: 1843–1848

Parkin DM, Whelan SC, Ferlay J, Raymond L and Young J (1997) *Cancer Incidence in Five Continents* Vol VII. IARC Scientific Publications 143, IARC: Lyon

Parsa R, Yang A, McKeon F and Green H (1999) Association of p63 with proliferative potential in normal and neoplastic human keratinocytes. *J Invest Dermatol* **113**: 1099–1105

Pisani P, Parkin DM, Bray F and Ferlay J (1999) Estimates of the worldwide mortality from 25 cancers in 1990 [published erratum appears in Int J Cancer 1999 Dec 10; 83(6):870–3]. *Int J Cancer* **83**: 18–29

Robert V, Michel P, Flaman JM, Chiron A, Martin C, Charbonnier F, Paillot B and Frebourg T (2000) High frequency in esophageal cancers of p53 alterations inactivating the regulation of genes involved in cell cycle and apoptosis. *Carcinogenesis* **21**: 563–565

Shamma A, Doki Y, Tsujinaka T, Shiozaki H, Inoue M, Yano M, Kawanishi K and Monden M (2000) Loss of p27(KIPI) expression predicts poor prognosis in patients with esophageal squamous cell carcinoma. *Oncology* **58**: 152–158

Shirvani VN, Ouatu-Lascar R, Kaur BS, Omary MB and Triadafilopoulos G (2000) Cyclooxygenase 2 expression in Barrett's esophagus and adenocarcinoma: Ex vivo induction by bile salts and acid exposure. *Gastroenterology* **118**: 487–496

Tanière P, Martel-Planche G, Maurici D, Lombard-Bohas C, Scoazec JY, Berger F and Hainaut P (2000a). Molecular and clinical differences between adenocarcinomas of the esophagus and of the gastric cardia. *Am J Pathol* **158**: 33–40

Taniere P, Martel-Planche G, Puttawibul P, Casson A, Montesano R, Chanvitan A and Hainaut P (2000b) TP53 mutations and MDM2 gene amplification in squamous-cell carcinomas of the esophagus in South Thailand. *Int J Cancer* **88**: 223–227

Tudek B, Kowalczyk P and Ciesla JM (1999) Localization of chloroacetaldehyde-induced DNA damage in human p53 gene by DNA polymerase fingerprint analysis. IARC Sci Publ 150, pp 279–293

Tuyns AJ (1987) Cancer risks derived from alcohol. *Med Oncol Tumor Pharmacother* **4**: 241–244

Wang QS, Sabourin CL, Wang H and Stoner GD (1996) Overexpression of cyclin D1 and cyclin E in N-nitrosomethylbenzylamine-induced rat esophageal tumorigenesis. *Carcinogenesis* **17**: 1583–1588

Yamaguchi K, Wu L, Caballero OL, Hibi K, Trink B, Resto V, Cairns P, Okami K, Koch WM, Sidransky D and Jen J (2000) Frequent gain of the p40/p51/p63 gene locus in primary head and neck squamous cell carcinoma. *Int J Cancer* **86**: 684–689

Yang A, Kaghad M, Wang Y, Gillett E, Fleming MD, Dotsch V, Andrews NC, Caput D and McKeon F (1998) p63, a p53 homolog at 3q27–29, encodes multiple products with transactivating, death-inducing and dominant-negative activities. *Mol Cell* **2**: 305–316

Yang A, Schweitzer R, Sun D, Kaghad M, Walker N, Bronson RT, Tabin C, Sharpe A, Caput D, Crum C and McKeon F (1999) p63 is essential for regenerative proliferation in limb, craniofacial and epithelial development. *Nature* **398**: 714–718

Zambon P, Talamini R, La Vecchia C, Dal Maso L, Negri E, Tognazzo S, Simonato L and Franceschi S (2000) Smoking, type of alcoholic beverage and squamous-cell oesophageal cancer in northern Italy. *Int J Cancer* **86**: 144–149

 *© 2001 Cancer Research Campaign*
